# Effectiveness of mood stabilizers in prophylactic treatment of bipolar disorder

**DOI:** 10.1192/j.eurpsy.2023.1455

**Published:** 2023-07-19

**Authors:** D. Khattech, A. Aissa, U. Ouali, Y. Zgueb, R. Jomli

**Affiliations:** Razi Hospital, Manouba, Tunisia

## Abstract

**Introduction:**

Prophylactic treatment during bipolar disorder aims to prevent recurrences and to improve the functional level.

**Objectives:**

Our aim was to compare the clinical effectiveness of lithium versus sodium valproate in the prophylactic treatment of bipolar disorder type 1

**Methods:**

Retrospective, longitudinal, comparative study conducted among 162 patients followed for bipolar disorder type 1 hospitalized at the Psychiatry A department of Razi Hospital. The Alda scale and time to recurrence were used to compare the clinical effectiveness of the mood stabilizers.

**Results:**

A difference between the two groups of patients was noted for some variables. Lithium prescription was associated with educational level, number of depressive episodes, suicide attempts, previous prescription of other thymoregulators, depressive polarity of the index episode and use of atypical antipsychotics.

The prescription of Valproate was associated with educational level, unipolar mania, manic predominant polarity, manic polarity of the index episode, presence of psychotic features, prescription of long acting antipsychotics and higher doses of antipsychotics.

The study of response by Alda scale showed no significant difference in the mean score of the scale nor in the rate of responders. We noted a higher rate of recurrence in patients on Valproate considering the whole duration of the study. The recurrence rate after one year was higher in patients on Lithium, the recurrence rate after two years was comparable in both groups. Survival curves showed earlier recurrences in patients on Lithium.

**Conclusions:**

The efficacy of the two mood stabilizers was comparable. The recurrences occured earlier under Lithium.

**Disclosure of Interest:**

None Declared

